# Interaction between birds and macrofauna within food webs of six intertidal habitats of the Wadden Sea

**DOI:** 10.1371/journal.pone.0176381

**Published:** 2017-05-10

**Authors:** Sabine Horn, Camille de la Vega, Ragnhild Asmus, Philipp Schwemmer, Leonie Enners, Stefan Garthe, Kirsten Binder, Harald Asmus

**Affiliations:** 1 Alfred-Wegener-Institut Helmholtz-Zentrum für Polar- und Meeresforschung, WaddenSea Station Sylt, List/ Sylt, Germany; 2 Research and Technology Centre (FTZ), University of Kiel, Büsum, Germany; 3 State Agency for Agriculture, Environment and Rural Areas Schleswig-Holstein, Flintbek, Germany; University of Waikato, NEW ZEALAND

## Abstract

The determination of food web structures using Ecological Network Analysis (ENA) is a helpful tool to get insight into complex ecosystem processes. The intertidal area of the Wadden Sea is structured into diverse habitat types which differ in their ecological functioning. In the present study, six different intertidal habitats (i.e. cockle field, razor clam field, mud flat, mussel bank, sand flat and seagrass meadow) were analyzed using ENA to determine similarities and characteristic differences in the food web structure of the systems. All six systems were well balanced between their degree of organization and their robustness. However, they differed in their detailed features. The cockle field and the mussel bank exhibited a strong dependency on external imports. The razor clam field appeared to be a rather small system with low energy transfer. In the mud flat microphytobenthos was used as a main food source and the system appeared to be sensitive to perturbations. Bird predation was the most pronounced in the sand flat and the seagrass meadow and led to an increase in energy transfer and parallel trophic cycles in these habitats. Habitat diversity appears to be an important trait for the Wadden Sea as each subsystem seems to have a specific role in the overall functioning of the entire ecosystem.

## Introduction

The World Heritage Site of the Wadden Sea is one of the world’s most valuable stretches of coastline [1]. It consists of vast bare sand and mud flats that emerge twice per day during low tide forming a unique ecosystem [1, 2]. The highly productive intertidal areas are characterized by a rich benthic fauna supporting millions of coastal birds that visit the Wadden Sea for foraging, resting or breeding on the East Atlantic Flyway [1–4].

The interaction of physical forces and biological activities turn the extensive intertidal flats into heterogeneous habitats either represented by differences in their sediment characteristics or in their dominant species aggregation [2]. This heterogeneity is an important requirement for different macrobenthic species to find a settling ground as well as for higher predators such as birds or fish that might be specialized to forage in a certain environment.

However, little is known about the ecological functioning of the different habitat types and their role in the ecosystem of the Wadden Sea. Food web modeling and especially Ecological Network Analysis (ENA) are appropriate tools to gain insight into the complexity of system structures. Indeed, ENA accounts for the totality of the interactions between the various components of the system [5]. It allows a simplified representation of the natural system based on flows of energy between different feeding levels [6]. The methodology was developed to assess the complex interactions within an ecosystem using a set of algorithms from which several system properties can be derived [7, 8]. Results from ENA provide information that can be used to assess environmental issues but also to describe the system’s status in terms of maturity, health, stability and stress [7, 8].

There were already several approaches to describe intertidal areas using ENA. The food web of the Sylt-Rømø Bight in the northern Wadden Sea was already intensively studied in different energy units and differences in the recycling of carbon, nitrogen and phosphorus have been found in this tidal basin [9–12]. Furthermore, invasive species are known to have settled in the Wadden Sea (e.g. Sylt-Rømø Bight) which couldhave changed the trophic functioning of the system [13]. Schückel, Kröncke [8] described the benthic food web of the Jade-Bay (south-eastern Wadden Sea) from the 1930s to the present status and found differences in the functioning of the bay probably caused by climatic changes and anthropogenic impacts such as eutrophication. However, food web studies focusing on birds are very rare as birds are difficult to include in quantitative models due to their mobility. In the French Marénnes-Oléron Bay the influence of migratory shorebirds on the food web structure of mud flats was shown by Saint-Béat, Dupuy [14] by regularly counting the birds feeding in the bay. But in the majority of cases, roosting bird data from the coastline is used for modeling [10, 11] that is then interpolated to the intertidal areas. The bird numbers therefore often underly large approximations as it is not known in which habitats the birds prefer to feed.

In the present study, the structure and functioning of different intertidal habitats was studied in a modeling approach including foraging birds as top predators. The study site is situated between several islands that are known to be important breeding and resting places for various bird species which take up food on the intertidal flats [2]. Despite its importance for birds, the area is only rarely studied and differs from already investigated intertidal areas in terms of its connection to the open North Sea and its habitat heterogeneity. The main goals of this study were 1) to create food web models of six different habitats in the Wadden Sea that are known to be strongly used by foraging birds and 2) to determine the similarities and differences in the functioning of the distinct systems to find characteristic features for the habitat types.

## Materials and methods

### Ethical statement

Sampling in the study site was approved by the National Park Authority, Tönning within the framework of the project “STopP—From sediment to top predator” (project number BMBF, FKZ, 03F672B).

In the Wadden Sea, coastal birds are highly protected under comprehensive regulations and conventions such as the EU Birds Directive (1979), the Species of European Conservation Concern (2004), the Bonn Convention on the Conservation of Migratory Species of Wild Animals (1979), the Bern Convention (Convention on the Conservation of European Wildlife and Natural Habitats, 1979) and the Agreement on the Conservation of African-Eurasian Migratory Waterbirds (1995)[15]. Therefore, all data concerning birds were referred to observations and the birds were not disturbed in their natural behavior.

### Study site

**Fig 1:**
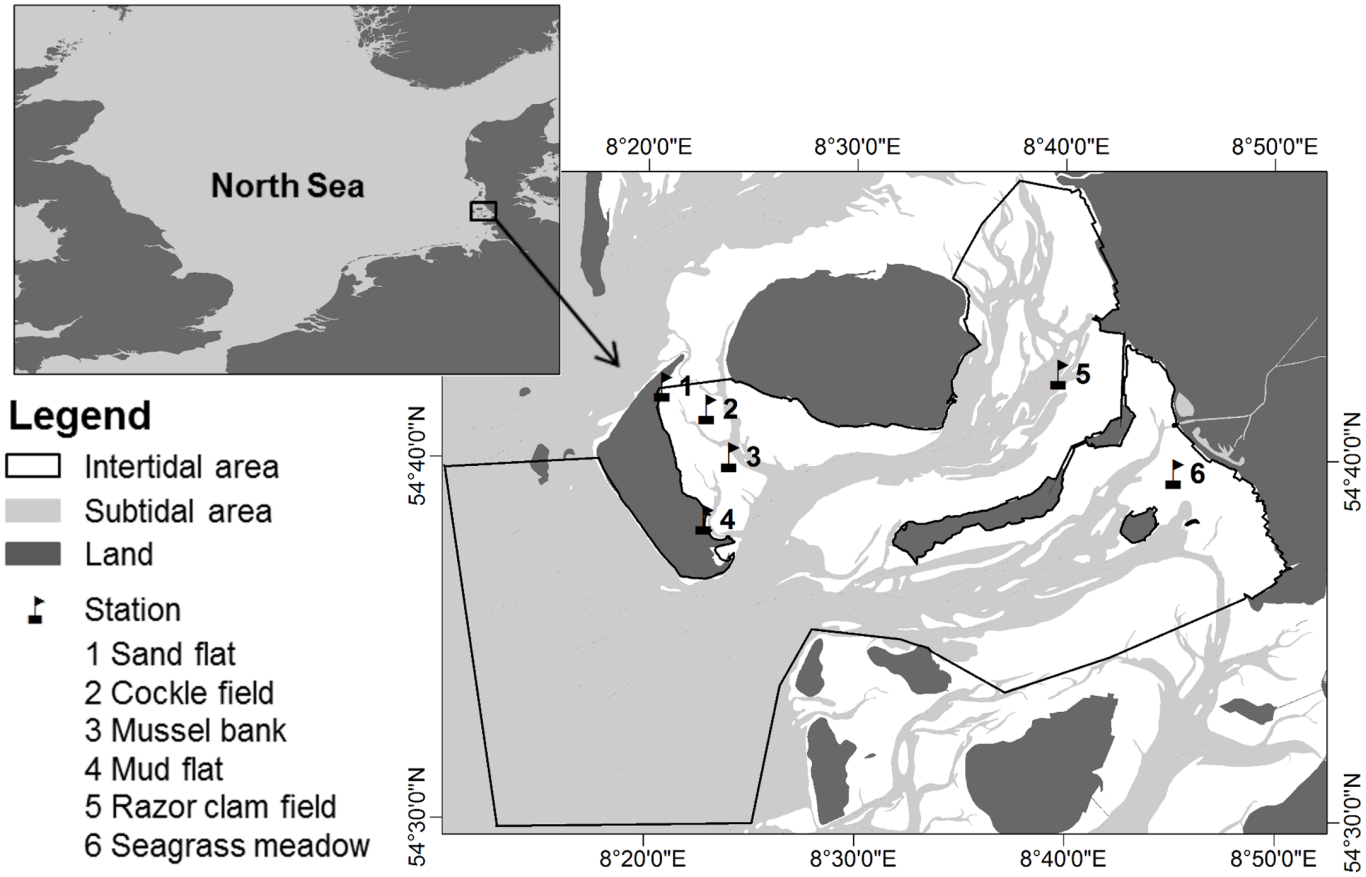
Location of the study site in the north-eastern Wadden Sea. The black frame delimits the study site. Sampling locations in the different habitat types are represented by black flags, map source: Topographic GIS map 2003, issued by National Park Authority, Tönning.

Samples for network construction were collected from summer 2013 to summer 2015 in the German part of the Wadden Sea between the islands Amrum, Föhr, Langeness and the western coast of the federal state of Schleswig-Holstein (Fig 1). The study site had a total size of 655.4 km^2^ with an intertidal area of 286.3 km^2^. Six different habitats of the intertidal area (i.e. cockle field, razor clam field, mud flat, mussel bank, sand flat and seagrass meadow) were either defined by their sediment type (i.e. mud flat and sand flat) or by their dominating species (i.e. cockle field, razor clam field, mussel bank and seagrass meadow).

Cockle fields cover about 6.3 km^2^ of the area and are characterized by a very high abundance of the common cockle *Cerastodermaedule* which can reach densities up to thousand individuals per m^2^ [16]. A rather new habitat are the razor clam fields that are formed by aggregations of the immigrant American razor clam *Ensisleei* and are located in wide parts of the lower intertidal (31.5 km^2^) and subtidal areas of the study site. Mud flats are soft bottom habitats and occur in sheltered areas with low current velocities close to the shore. About 23.1 km^2^ of the area are mud flats (Brockmann Consult GmbH 2014, unpublished data). Mussel banks are small-scaled epibenthic structures dominated by the blue mussel *Mytilus edulis* mixed with the invasive Pacific oyster *Magallanagigas* since the late 1980s. Only 0.6 km^2^ of the study site represent mussel banks (Brockmann Consult GmbH 2014, unpublished data). Sand flats are the most extended habitat type in the study area with 160.3 km^2^ (Brockmann Consult GmbH 2014, unpublished data). They are often dominated by dense populations of the lugworm *Arenicola marina*. 33.3 km^2^ of the area are overgrown by seagrass meadows (Brockmann Consult GmbH 2014, unpublished data) dominated by the dwarf eelgrass *Zosteranoltei* with sparse occurrences of the common eelgrass *Zostera marina*.

A transect of 200 to 250 m length that included five to six sampling stations located in a distance of 50 m away from each other was placed in each habitat. Each station was covered by a 25x25 cm frame to define the area for quantitative sampling. The samples were taken seasonally to receive the required data for network construction.

### Sampling

In this study, only benthic components, phytoplankton and birds were sampled and included in analyses as the main focus of this study was the interaction between intertidal areas and foraging birds. Each species or group of species was represented by a compartment within the model (Table 1). Birds were the only modules of higher trophic levels in the analyzed models. Production used by other predators (i.e. fish, seals) is therefore included in the export of unused production from prey compartments.

**Table 1:** Standing stocks in mgC m^-2^ of the compartments for the six habitats, energy ratios applied to calculate the energy budget of each compartment in mgC m^-2^ d^-1^ and references of the energy ratios.

Compartment	Cocklefield	Razorclamfield	Mud flat	Musselbank	Sand flat	Seagrassmeadow	Source ofdata	GPP/B	R/B	NPP/B		Source ofratios
Phytoplankton	605.83	706.80	468.51	605.83	468.51	468.51	Long-term data (LLUR)	0.4205	0.1828	0.2378		[11]
Macrophyta	428.02	-	-	105,400.21	2,855.20	3,819.14	Presentstudy	0.0274	0.0153	0.0121		Baird, Asmus [11]
MPB	15,399.63	10,780.72	29,646.11	14,665.87	9,164.13	7,560.45	Presentstudy	0.300	0.0825	0.2175		Degré, Leguerrier [31]
								**P/B**	**R/B**	**E/B**	**C/B**	
BAC	625.00	625.00	625.00	625.00	625.00	625.00	[11]	0.0788	0.1744	0.0610	0.3924	Baird, Asmus [10], Baird, Asmus [11]
MEI	1,000.00	1,000.00	500.00	500.00	1,000.00	1,000.00	[11]	0.0219	0.0834	0.1832	0.2885	[10, 11]
*Anthozoa*	-	-	-	6,860.88	-	-	Presentstudy	0.0023	0.0087	0.0013	0.0123	[32], [11]
*Cerastodermaedule*	129,451.19	-	15,709.57	13,056.26	3,942.88	3,363.42	Presentstudy	0.0050	0.0016	0.0184	0.0249	[11]
*Ensisleei*	-	12,355.62	-	-	-	-	Presentstudy	0.0065	0.0206	0.0014	0.0285	[33]
*Fabulinafabula*	-	6.82	-	-	-	-	Presentstudy	0.0082	0.0015	0.0435	0.0533	[11]
*Limecolabalthica*	6,832.16	1,184.65	1,134.98	6.47	1,811.77	730.11	Presentstudy	0.0082	0.0015	0.0435	0.0533	[11]
*Magallanagigas*	-	-	-	42,450.47	-	-	Presentstudy	0.0010	0.0130	0.0008	0.0148	[11]
*Myaarenaria*	408.12	-	-	-	1,792.66	-	Presentstudy	0.0022	0.0051	0.0037	0.0109	[11]
*Mytilusedulis*	199.79	-	-	292,734.20	495.78	-	Presentstudy	0.0010	0.0054	0.0009	0.0073	[11]
*Balanidaespp*.	-	-	-	24,184.15	-	7.95	Presentstudy	0.0033	0.0087	0.0013	0.0133	[9]
*Carcinus+Hemigrapsus*	345.10	-	6,890.40	12,332.60	-	93.06	Presentstudy	0.0042	0.0063	0.0139	0.0243	[11]
*Crangonspp*.	378.33	1,223.80	58.73	146.62	-	-	Presentstudy	0.0110	0.0378	0.0110	0.0598	[11]
*Pycnogonum litorale*	-	-	-	90.48	-	-	Presentstudy	0.0190	0.0268	0.0054	0.0265	[11]
*smallcrustaceans*	150.78	120.18	13.59	128.25	5.48	767.97	Presentstudy	0.0040	0.0171	0.0054	0.0265	[11]
*Crepidulafornicata*	-	-	-	287.38	-	-	Presentstudy	0.0009	0.0062	0.0124	0.0195	[32], [10]
*Lepidochitonacinerea*	-	-	-	283.50	-	-	Presentstudy	0.0050	0.0062	0.0124	0.0235	[11, 32]
*Littorinalittorea*	990.64	-	660.16	18,115.03	1,275.77	993.02	Present study	0.0020	0.0062	0.0124	0.0206	[11]
*Peringiaulvae*	11,186.09	0.81	17,248.54	29.67	17,514.95	11,914.42	Present study	0.0180	0.0060	0.0291	0.0532	[32], [11]
*Retusaobtusa*	33.10	2.38	36.02	-	1,124.62	28.94	Presentstudy	0.0039	0.0060	0.0291	0.0391	[11]
*Nemertea*	-	-	-	181.98	175.29	-	Presentstudy	0.0065	0.0105	0.0380	0.0549	[32], [11]
*Oligochaeta*	33.97	8.60	418.07	3,062.51	966.26	61.72	Presentstudy	0.0027	0.0267	0.0135	0.0736	[11]
*Arenicolamarina*	1,623.65	-	-	742.40	4,833.16	2,033.25	Presentstudy	0.0072	0.0067	0.0339	0.0478	[11]
*Capitellacapitata*	108.57	9.47	45.61	331.86	54.61	10.29	Presentstudy	0.0054	0.0231	0.0567	0.0850	[11]
*Eteonespp*.	70.64	7.54	7.37	37.06	44.13	64.49	Presentstudy	0.0048	0.0007	0.0084	0.0285	[11]
*Heteromastusfiliformis*	-	-	-	52.21	-	-	Presentstudy	0.0055	0.0104	0.0700	0.0859	[11]
*Laniceconchilega*	90.19	-	2.78	5,502.67	-	-	Presentstudy	0.0052	0.0100	0.0046	0.0199	[11]
*Lepidonotussquamatus*	-	-	-	376.30	-	-	Presentstudy	0.0033	0.0105	0.0380	0.0517	[32], [11]
*Nephtysspp*.	956.39	828.49	-	198.48	-	5.74	Presentstudy	0.0110	0.0105	0.0380	0.0595	[11]
*Hedistespp*.	1,450.88	-	9,243.83	1,294.15	455.63	382.76	Presentstudy	0.0048	0.0117	0.0307	0.0472	[34], [11]
*Phyllodocespp*.	259.84	-	-	601.32	704.44	-	Presentstudy	0.0027	0.0296	0.0039	0.0360	[11]
*Pygospioelegans*	45.80	16.52	24.26	4.41	29.49	57.43	Presentstudy	0.0037	0.0170	0.0073	0.0280	[11]
*Scoloplos armiger*	38.73	191.36	24.85	63.64	2,761.94	304.01	Presentstudy	0.0044	0.0073	0.0189	0.0306	[34], [11]
*smallpolychaetes*	55.16	34.16	38.34	13,507.19	4.93	5.34	Presentstudy	0.0045	0.0146	0.0084	0.0285	[11]
*Tharyxkillariensis*	25.15	-	80.73	6.53	1.86	91.98	Presentstudy	0.0055	0.0104	0.0111	0.0272	[11]
*Anas acuta*	-	-	-	-	-	223.79	Presentstudy	0.0029	0.0606	0.0346	0.0981	[11]
*Anas penelope*	-	-	-	-	2,883.24	3,592.84	Presentstudy	0.0006	0.0179	0.0104	0.0289	[11]
*Anas platyrhynchos*	-	-	33.85	7.58	254.81	348.33	Presentstudy	0.0006	0.0210	0.0125	0.0341	[11]
*Arenariainterpres*	-	-	-	1.12	5.99	5.55	Presentstudy	0.0018	0.1072	0.0277	0.1367	[11]
*Brantabernicla*	-	-	-	-	239.50	1,429.48	Presentstudy	0.0010	0.0250	0.0140	0.0400	[11]
*Calidrisalpina*	-	-	15.85	-	5.70	584.34	Presentstudy	0.0021	0.1198	0.0310	0.1528	[11]
*Calidriscanutus*	-	-	-	-	99.97	588.30	Presentstudy	0.0037	0.1963	0.0519	0.2519	[11]
*Charadriushiaticula*	-	-	-	-	5.33		Presentstudy	0.0021	0.1198	0.0310	0.1528	[11]
*Chroicocephalusridibundus*	23.43	3.86	94.23	1.63	31.01	165.59	Presentstudy	0.0023	0.0537	0.0140	0.0700	[11]
*Haematopusostralegus*	359.25	7.02	79.00	16.12	366.54	916.81	Presentstudy	0.0045	0.1040	0.0271	0.1357	[11]
*Larusagentatus*	50.22	95.10	39.37	7.67	124.39	136.35	Presentstudy	0.0015	0.0452	0.0121	0.0588	[11]
*Laruscanus*	23.49	5.01	18.40	6.46	56.25	24.99	Presentstudy	0.0018	0.0390	0.0106	0.0514	[11]
*Larusfuscus*	-	14.29	18.49	-	-	-	Presentstudy	0.0004	0.0117	0.0031	0.0152	[11]
*Larusmarinus*	-	19.60	-	-	-	-	Presentstudy	0.0006	0.0003	0.0078	0.0086	[11]
*Limicolafalcinellus*	-	-	-	-	-	1.80	Presentstudy	0.0021	0.1198	0.0310	0.1528	[11]
*Limosalapponica*	97.85	10.55	187.15	10.67	909.63	309.34	Presentstudy	0.0037	0.1593	0.0407	0.2037	[11]
*Numeniusarquata*	80.20	2.04	28.20	7.66	98.59	369.31	Presentstudy	0.0018	0.0551	0.0147	0.0716	[11]
*Numeniusphaeopus*	-	-	-	-		33.56	Presentstudy	0.0018	0.0551	0.0147	0.0716	[11]
*Pluvialissquatarola*	-	-	10.62	1.15	63.12	105.75	Presentstudy	0.0031	0.0875	0.0219	0.1125	[11]
*Recurvirostraavosetta*	-	-	39.82	-	-	-	Presentstudy	0.0111	0.1667	0.0444	0.2222	[11]
*Somateriamollissima*	38.95	6.81	89.72	206.54	-	-	Presentstudy	0.0027	0.1060	0.0271	0.1358	[11]
*Tadornatadorna*	384.99	-	95.91	-	290.08	94.79	Presentstudy	0.0024	0.0809	0.0241	0.1074	[11]
*Tringaerythropus*	-	-	-	-	-	41.99	Presentstudy	0.0036	0.1904	0.0503	0.2443	[11]
*Tringanebularia*	-	-	4.07	0.55	7.35	31.65	Presentstudy	0.0033	0.1767	0.0467	0.2267	[11]
*Tringatotanus*	187.19	-	81.47	4.38	58.08	139.89	Presentstudy	0.0036	0.1904	0.0503	0.2443	[11]
sediment POC	19,000.00	19,000.00	19,000.00	19,000.00	19,000.00	19,000.00	[11]					[10]
suspended POC	167.44	167.44	167.44	167.44	167.44	167.44	[11]					[10]

B = Biomass, GPP = Gross primary production, NPP = Net primary production, P = Production, R = Respiration, E = Egestion, C = Consumption

#### Macrobenthos

Epifauna and macrophytes within each of the 25x25 cm frames were removed from the surface by hand. Infauna was sampled with a 10x10 cm corer 15 cm deep and afterwards sieved through a 0.5 and 1 mm mesh-cascade. Samples were sorted and organisms were identified to the most precise taxonomic level and counted.

For biomass determination, each species of macrofauna and the macrophytes were dried in an oven at 50°C until constant dry weight. They were then burned at 500°C in a furnace for 5 h. Ash free dry weight (AFDW) was estimated by subtracting the ash weight from the dry weight and further transformed to mg Carbon (C) using the conversion factor 0.58 for invertebrates [17].

#### Microphytobenthos

Samples for microphytobenthos (MPB) were taken by outpacing the first cm of the sediment surface with a corer (ø 1 cm). The sediment was freeze-dried and Chlorophyll a content was measured following the protocol of Edler [18] and calculated according toJeffrey and Humphrey [19]. The Chlorophyll a content was multiplied by 50, to convert it to mg C [20].

#### Phytoplankton

Chlorophyll a data for phytoplankton was taken from a long-term monitoring program conducted monthly in the project area by the State Agency for Agriculture, Environment and Rural Areas of Schleswig-Holstein (LLUR). The data were converted to mg C by multiplying Chlorophyll a values by 50 [20].

#### Birds

Birds, except for eider ducks (*Somateriamollissima*), were counted depending on weather conditions one to three times per season in each habitat in a predefined area of 0.01 km^2^ (cockle field) to 0.16 km² (mussel bank) and identified to species level. Counts occurred in 10 min intervals for 2 h. Only the abundance of foraging birds was included in the analyses.

Eider duck data were taken from regular aerial counts. The abundance of eider ducks was interpolated to the habitat types they feed on (i.e. mussel bank, cockle field, razor clam field) using the total size of the habitats in the study site and the time the eider ducks spend feeding on the habitat type according to their diet composition.

It was assumed that birds of different size classes were distributed equally in the habitat types. Abundance of the bird data was therefore transformed to biomass using average body fresh weight values for each species (FTZ, unpublished data,[21]) and then converted into carbon units [22].

#### Additional data

In the study site no data were available for particulate organic carbon in the sediment (sediment POC), suspended particulate organic carbon in the water column (suspended POC), meiofauna (MEI) and bacteria (BAC). To create more realistic food web models these compartments were included in the network using data from similar habitats of the Sylt-Rømø Bight [10].

### Network construction

The construction of an ecological network requires information about the standing stock and energy budget of each compartment and about flows between compartments (i.e. who eats whom at what rate?[23]).

The determination of standing stock data is described above. Averaged values have been used for network construction (Table 1). Energy budgets were taken from recent published and unpublished literature and are summarized with references in Table 1. Diet information for benthic compartments were taken from Baird, Asmus [11]. Each compartment was balanced in terms of its energy budget following the equations of Parsons, Takahashi [24]

Gross primary production=Net primary production+Respiration

Consumption=Production+Respiration+Egestion

Several bird species feed on both intertidal areas and terrestrial environments but also on prey items that were not included in the present study such as fish. For those species (i.e. *Anasacuta*, *Anaspenelope*, *Anasplathyrhynchos*, *Arenariainterpres*, *Brantabernicla*, *Charadriushiaticula*, *Chroicocephalusridibundus*, *Haematopusostralegus*, *Larusargentatus*, *Laruscanus*, *Larusfuscus*, *Larusmarinus*, *Numeniusarquata*, *Numeniusphaeopus*, *Tadornatadorna*), the energy budget was adapted and the consumption value was decreased from 100% to the estimated percentage of time the birds spend feeding on intertidal flats. The diet matrix of the birds is given in Table 2. If a prey item of the diet spectrum of a particular bird species was not available in one of the habitats, the missing consumption flux was equally distributed to the available prey items.

**Table 2:** Diet matrix of the birds with references, numbers show the percentage contribution of each prey compartment i to the diet of each bird species (consumer compartment j), with A. a = *Anasacuta*, A. pe = *Anaspenelope*, A. pl = *Anasplaythyrhynchos*, A. in = *Arenariainterpres*, B. be = *Brantabernicla*, C. al = *Calidrisalpina*, C. ca = *Calidirscanutus*, Ch. hi = *Charadriushiaticula*, Ch. ri = *Chroicosephalusridibundus*, H. ost = *Haematopusostralegus*, L. arg = *Larusargentatus*, L. ca = *Laruscanus*, L. fu = *Larusfuscus*, L. ma = *Larusmarinus*, L. fa = *Limicolafalcinellus*, L. lap = *Limosalapponica*, N. arqu = *Numeniusarquata*, N. phae = *Numeniusphaepus*, P. squa = *Pluvialissquatarola*, R. avo = *Recurvirostraavosetta*, So. mo = *Somateriamollissima*, Ta. ta = *Tadornatadorna*, T. ery = *Tringaerythropus*, T. ne = *Tringanebularia*, T. to = *Tringatetanus*, FTZ data in the reference list refer to unpublished data from the Research and Technology Centre (FTZ) of the University of Kiel.

#	Consumer compartment j																								
Prey compartment i	A. a	A. pe	A. pl	A. ni	B. be	C. al	C. ca	Ch. hi	Ch. ri	H. ost	L. arg	L. ca	L. fu	L. ma	L. fa	L. lap	N. arqu	N. phae	P. squa	R. avo	So. mo	Ta. ta	T. ery	T. ne	T. to
Makrophyta	0.25	0.27	0.250	-	0.500	-	-	0.001	-	-	-	-	-	-	-	-	-	-	0.019	-	-	0.030	-	-	-
Anthozoa	-	-	-	-	-	-	-	-	-	-	-	-	-	-	-	-	-	-	-	-	-	-	-	-	-
*Cerastodermaedule*	-	-	-	0.280	-	-	0.517	-	0.070	0.300	0.200	0.050	0.030	-	-	-	0.150	0.150	-	-	0.210	0.030	0.040	0.040	0.040
*Crassostreagigas*	-	-	-	-	-	-	-	-	-	-	-	-	-	-	-	-	-	-	-	-	-	-	-	-	-
*Ensis directus*	-	-	-	-	-	-	-	-	-	0.100	0.150	-	0.060	0.100	-	-	-	-	-	-	0.100	-	-	-	-
*Fabulinafabula*	-	-	-	-	-	-	-	-	-	-	-	-	-	-	-	-	-	-	-	-	-	-	-	-	-
*Macomabalthica*	0.009	-	0.019	-	-	-	0.250	0.032	0.050	0.100	0.010	-	-	-	-	0.033	0.050	0.050	0.004	-	-	0.090	0.050	0.050	0.050
*Myaarenaria*	0.007	-	0.008	-	-	-	-	-	0.005	-	-	0.026	-	-	-	-	-	-	-	-	-	-	-	-	-
*Mytilusedulis*	-	-	-	0.280	-	-	0.043	-	-	0.100	0.010	0.010	0.030	-	-	-	-	-	-	-	0.328	0.025	-	-	-
Balanidae	-	-	-	-	-	-	-	-	-	-	-	-	-	-	-	-	-	-	-	-	0.020	-	-	-	-
*Carcinusmaenas*	0.005	-	0.006	0.043	-	-	-	0.020	0.020	-	0.200	0.040	-	-	-	0.015	0.200	0.200	0.003	0.100	-	0.010	0.100	0.100	0.100
*Crangoncrangon*	-	-	0.006	-	-	-	-	-	0.100	-	-	0.080	-	-	-	0.016	0.050	0.050	-	0.010	-	-	0.030	0.030	0.030
*Pygnogonumlittorale*	-	-	-	-	-	-	-	-	-	-	-	-	-	-	-	-	-	-	-	-	-	-	-	-	-
smallcrustaceans	0.010	-	0.058	0.004	-	0.143	-	0.020	0.010	-	-	0.025	-	-	0.143	-	-	-	0.003	0.010	-	0.080	0.300	0.300	0.300
*Crepidulafornicata*	-	-	-	-	-	-	-	-	-	-	-	-	-	-	-	-	-	-	-	-	0.010	-	-	-	-
*Lepidochitonacinerea*	-	-	-	-	-	-	-	-	-	-	-	-	-	-	-	-	-	-	-	-	-	-	-	-	-
*Littorina littorea*	-	-	-	0.120	-	-	-	0.030	-	-	-	-	-	-	-	-	-	-	0.004	-	0.155	0.095	-	-	-
*Peringiaulvae*	0.279	-	0.120	0.120	-	0.087	0.190	0.030	0.020	-	-	0.005	-	-	0.087	-	-	-	0.004	0.370	0.177	0.450	0.150	0.150	0.190
*Retusaobtusa*	-	-	-	-	-	-	-	-	0.005	-	-	-	-	-	-	-	-	-	-	-	-	0.050	-	-	-
Nemertea	-	-	-	-	-	-	-	-	-	-	-	-	-	-	-	-	-	-	-	-	-	-	-	-	-
Oligochaeta	0.019	-	0.003	-	-	-	-	-	-	-	-	-	-	-	-	-	-	-	-	0.010	-	-	-	-	-
*Arenicolamarina*	-	-	-	-	-	-	-	0.040	0.060	0.150	-	0.010	-	-	-	0.033	0.350	0.350	0.028	-	-	-	-	-	-
*Capitellacapitata*	0.019	-	0.003	-	-	-	-	0.250	-	-	-	-	-	-	-	-	-	-	0.245	-	-	-	-	-	-
*Eteonelonga*	-	-	-	-	-	-	-	-	-	-	-	-	-	-	-	-	-	-	-	-	-	-	-	-	-
*Heteromastusfiliformis*	0.019	-	0.003	-	-	0.050	-	0.250	0.010	-	-	0.010	-	-	0.050	-	-	-	0.245	-	-	-	-	-	-
*Laniceconchilega*	0.019	-	0.003	-	-	0.020	-	-	0.050	-	-	0.010	-	-	0.020	0.029	0.050	0.050	-	-	-	-	-	-	-
*Lepidonotussquamatus*	-	-	-	-	-	-	-	-	-	-	-	-	-	-	-	-	-	-	-	-	-	-	-	-	-
*Nephthyshombergi*	0.019	-	0.003	-	-	0.100	-	-	-	0.050	0.005	0.010	-	-	0.100	0.216	0.050	0.050	-	-	-	-	0.060	0.060	0.060
*Nereisdiversicolor*	0.019	-	0.003	0.003	-	0.500	-	0.030	0.090	0.100	0.005	0.120	0.030	-	0.500	0.276	-	-	0.200	0.500	-	0.080	0.170	0.170	0.200
*Phyllodocespp*.	0.019	-	0.003	-	-	-	-	-	-	-	-	-	-	-	-	-	-	-	-	-	-	-	-	-	-
*Pygospioelegans*	0.019	-	0.003	-	-	0.050	-	-	-	-	-	-	-	-	0.050	-	-	-	-	-	-	-	-	-	-
*Scoloplos armiger*	0.019	-	0.003	-	-	-	-	0.250	-	0.050	-	0.010	-	-	-	0.382	-	-	0.245	-	-	-	-	-	-
*smallpolychaetes*	0.019	-	0.003	-	-	-	-	-	-	-	-	-	-	-	-	-	-	-	-	-	-	-	-	-	-
*Tharyxkillariensis*	-	-	-	-	-	-	-	-	-	-	-	-	-	-	-	-	-	-	-	-	-	-	-	-	-
Fish/offshore prey	-	-	-	-	-	0.50	-	-	0.010	-	0.020	0.060	0.300	0.800	0.50	-	-	-	-	-	-	-	0.100	0.100	0.030
Terrestrial	0.250	0.730	0.500	0.150	0.500	-	-	0.050	0.500	0.050	0.400	0.550	0.550	0.100	-	-	0.100	0.100	-	-	-	0.060	-	-	-
**Reference**	[35–37]	[38]	[13, 35–37]	[39, 40]	[38]	[41]	[42]	[43]	[44, 45]	[46, 47]	FTZ data	[44], FTZ data	[44]	FTZ data	diet of*C*. *al*	[48]	[46]	diet of N. arqu	[49]	FTZ data	FTZ data	[50]	after [51]	after [51]	[51]

For each of the six habitats a carbon flow model was constructed. Biomass data was expressed in mgCm^-2^ and respiration, egestion and flows between compartments (i.e. production and consumption rates) as well as imports and exports to and from compartments were given in mgCm^-2^ d^-1^.

Number of compartments ranged from 29 in the razor clam field to 48 in the mussel bank. The difference in the number of compartments was due to the restriction of some species to single habitats and not due to a different degree of aggregation between the systems that might have biased comparisons of the ENA indices [25–29]. It was shown that an artificial homogenizing of system structure with zero-valued compartments might influence the results as well [30]. Therefore, we decided to represent the six habitats as they occurred in nature and tolerated the discrepancy in the number of compartments.

The total input of each compartment was balanced by the total output. If consumption of a compartment exceeded the production of a compartment of the preceding trophic level, an input was added to this compartment to fulfill the predator’s needs. Since this happened mostly due to bird predation it was assumed that the imported prey was consumed outside of the defined habitat, a plausible modus for mobile predators such as birds. Unused production was considered to be exported to one half as prey for compartments not included in this study such as fish or via resuspension during next high tide in terms of MPB. The other half was assumed to become sediment POC and flew back to the system. For phytoplankton, suspended POC and birds, the unused production was completely exported. Excess sediment POC was assumed to be exported from the system due to tidal flushing during storm events in the course of the year [10].

All six models therefore represented systems in steady-state. SCOR-files of all six network models are available as supporting information (S1–S6 Files).

### Network analysis

The methodology of Ecological Network Analysis is based on an input-output-analysis and is detailed in Kay, Graham [52] and reviewed by Ulanowicz [53]. In this study the software package enaR for R statistics was used to conduct all the analyses [54, 55]. ENA provides several helpful tools to describe the functioning and organization of an ecosystem. One of these tools is the system attributes. A collection of various global system indices describes the developmental and organizational state [8] but also the cycling and resilience of a system. The following indices were analyzed and described in the present study:

1. **Total System Throughput (TST):** The TST is the sum of all flows in the system and represents the system’s size and activity [56]. The higher the value the bigger and more active is the system.
2. **Ascendency (A):** It is a measurement for the system’s size and the flow organization and a natural descriptor of the combined processes of growth and development [56, 57]. High values are furthermore an indication for a high degree of specialization in the system.
  The **Relative Ascendency (A/DC)** is the ratio between A and DC and represents the system’s degree of organization and the efficiency of energy flows. A high A/DC shows a well-organized and developed system [56, 58].
3. **Overheads (OH):** The overheads characterize the free energy in a system. One part of the overhead is generated by three separate components of exogenous transfer; the inputs, the exports and the dissipation [56]. But also parallel flows in the internal exchanges, the redundancy, contribute to the overhead [11, 57]. With a high overhead the system has more capacities to react to perturbations and a larger potential of resilience. The ratio between OH and DC is described as the **Relative Overheads (OH/DC)** which is thenatural counterpart of A/DC.
4. **Development Capacity (DC):** This value describes the system’s potential to develop. It calculates a particular set of connections by multiplying the Total System Throughput with the diversity of individual flows [7]. Furthermore, it represents the sum of the system’s Ascendency and the system’s Overhead and is therefore the upper limit of the system’s Ascendency [56, 57].
5. **Robustness:** It is a measurement for the system’s sustainability. A high value shows more stable energy flows that are less sensitive to external disturbances [59, 60].
6. **Gross primary production *versus* biomass (P_GPP_/B):** This ratio is a function of the system’s maturity. It is expected that biomass is accumulated when the system matures. Therefore, the value decreases with system’s maturity [61, 62].
7. **Flow Diversity (FD):** It is a measurement for the number of interactions and the evenness of energy flows [56] and is defined as DC/TST [57]. Comparable to the biodiversity index, a high value shows a highly diverse and well-developed system [56, 58].
8. **Effective Link-Density (ELD):** It is the effective number of parallel pathways in the structure and is based on the mean number of flows per node [63].
9. **Average Path Length (APL):** It measures the mean number of compartments a unit of carbon passes from when it enters to the moment it leaves the system [9, 56, 64, 65]. The APL is supposed to rise under normal succession [8] and can therefore also be an indication for increased system’s maturity [58, 61].
10. **Finn Cycling Index (FCI):** This index shows the proportion of flows in a system that are recycled [56, 65]. High values indicate that the system is more independent from imports.
11. **Trophic Efficiency (TE):** The TE represents the efficiency of energy transfer from one trophic level to the next [11]. For the entire system the TE is calculated as the logarithmic mean of the integer trophic levels [7, 10, 11, 14].
12. **Trophic Depth (TD):** It is the effective number of trophic levels in the system and represents the number of functional roles in the food web [63].

The Lindeman Trophic Aggregation Analysis is another helpful implementation that transforms the complex food web network into a linear food chain (i.e. the Lindeman spine) with integer trophic levels [56]. In this representation all primary producers and the detritus pool form the first trophic level and consumers are distributed in the following trophic levels according to their feeding behavior. The Lindeman spine shows the amount of carbon each trophic level receives from the previous one as well as energy losses due to respiration and exports. It provides a quantitative estimation of the efficiency of the energy transfer within the system. The analysis also allows a comparison of the relation between detritivory and herbivory in a system which can be expressed as the ratio Detritivory: Herbivory.

### Uncertainty analysis

The models are based on empirical data, which can show natural variations in space (e.g. biomass variation of some species in patchy areas) or in time (e.g. seasonal variation of some species’ diets or seasonal and diurnal abundance of mobile predators such as birds). Therefore, a percentage of variation can be defined for each of the flows in the network. In this study, we conducted an uncertainty analysis for all six habitats in order to test the sensibility of the ENA indices to changes in the network parameters. The analysis was based on changes in the magnitude of flows in the network systems by 50%. We applied a modified version of the enaUncertainty approach within enaR which was initiated by Hines, Lisa [66], Hines, Singh [67]. The approach is based on linear inverse modeling and a Monte Carlo approach to generate additional plausible models [66–68] using the limSolve package in R [69–71]. For each system, 1,000 plausible, balanced network models were generated which differed randomly in their flow structure up to ± 50% from the initial model. System indices were calculated for all generated network models. Flow shifts of 50% represent severe changes in an ecosystem and thus could give an appropriate overview of the respective index’s variation. A difference between the indices was considered to be significant if the 95% confidence intervals did not overlap.

## Results

### Model evaluation

The quality evaluation of the assessed models is based on the data score ranking of Costanza, Funtowicz [72]. Field data and direct measurements are of high quality while data which is based on calculations is of medium quality. Estimations represent low quality data [73, 74]. Most of the data used in the present study is therefore of high or medium quality as they are received from field data (e.g. standing stock data), publications which are based in measurements (e.g. energy ratios) or calculations. Some data sources are of low quality such as the flows which are based on rough assumptions (e.g. exports and flows to detritus of unused production) or the flows estimated by mass-balancing (e.g. additional imports). These low quality flows might be over- or underestimated, that might influence the results related to cycling and recycling indices. Additional data are strongly needed to evaluate more precisely these fluxes.

### System functioning

#### Cockle field

**Fig 2:**
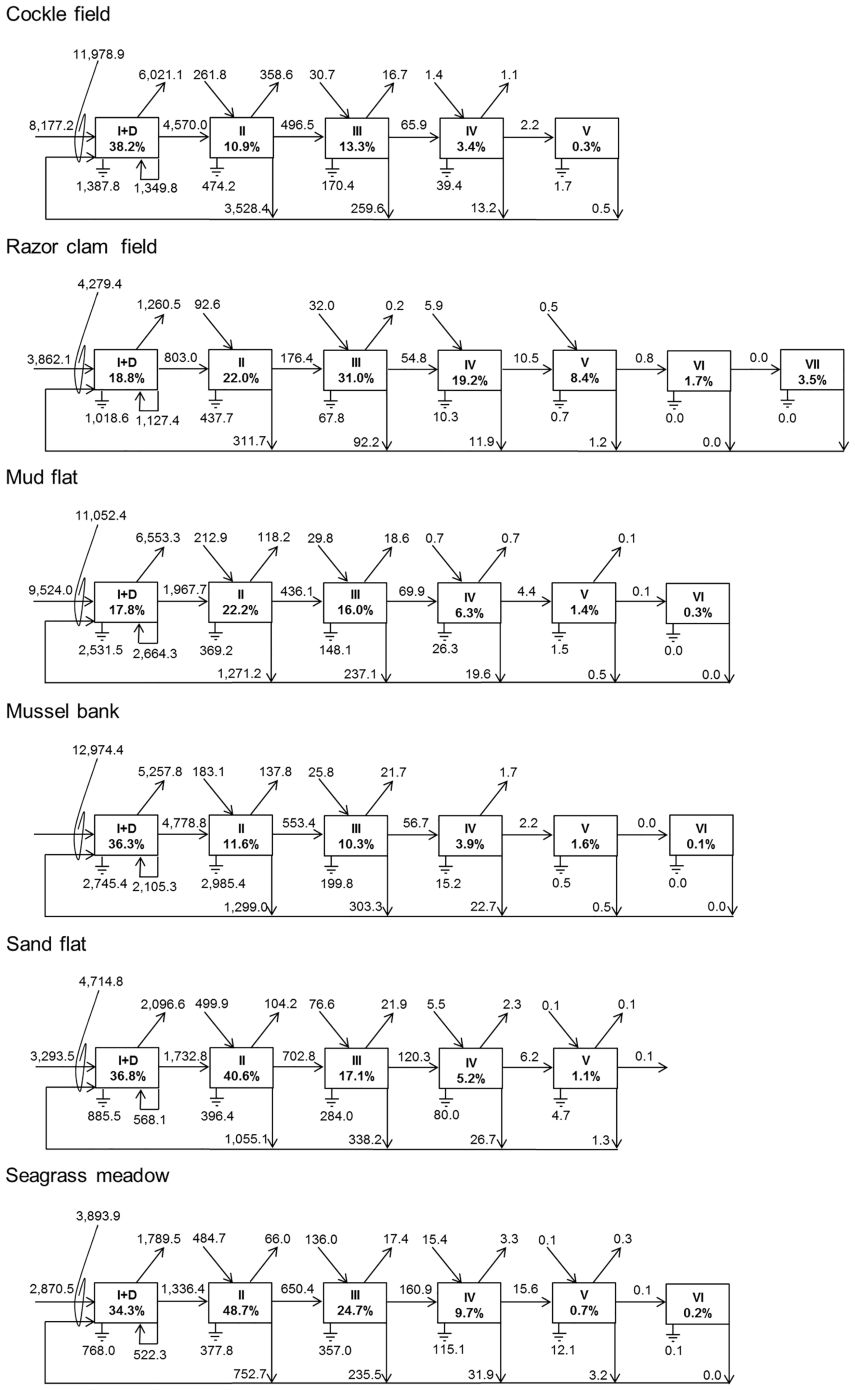
Lindeman spines of the six intertidal systems. Boxes represent the distinct trophic levels, percentage values refer to trophic efficiency between the levels. Arrows indicate energy flows between trophic levels as well asimports, exports and flows to detritus. Ground symbols show energy losses due to respiration. Values are given in mgC m^-2^ d^-1^.

The cockle field stores and provides a high amount of energy with a total biomass of 178,227.1 mgC m^-2^ and total production of 1,019.6mgC m^-2^ d^-1^ (Table 3). The habitat is a big and productive system with a high potential to further development indicated by a high TST of 27,228.2 mgC m^-2^ d^-1^ and a high DC of 111,005.2 mgC m^-2^ d^-1^. In accordance with the low recycling (FCI = 2.0%) the system is highly dependent on external imports resulting in an increased sensitivity to external perturbations. This sensitivity could also be supported by the high degree of organization with an A/DC of 43.2% which indicates a high amount of specialized flows. However, energy is transferred efficiently from the basis of the food web to higher trophic levels (Fig 2). In comparison to the other systems, the cockle field reveals a high APL of 2.2 implying that the energy is used in several compartments by different functional groups (TD = 3.4). The low P_GPP_/B value indicates a high system’s maturity [61] showing that the system is in a well-developed status.

**Table 3:** System attributes of the six intertidal systems.

System Attributes	Cocklefield	Razorclamfield	Mud flat	Musselbank	Sand flat	Seagrassmeadow
Numberofcompartments	38	29	38	48	43	45
Numberoflivingcompartments	36	27	36	46	41	43
total Biomass [mg C m^-^²]	193,205.8	48,434.7	102,881.0	577,904.6	76,775.5	62,700.9
total Production [mg C m^-^² d^-1^]	4,518.3	2,698.9	7,098.8	5,371.6	2,636.3	2,151.0
secondary Production [mg C m^-^² d^-1^]	1,019.6	186.0	539.4	767.2	426.1	349.2
total Exports [mg C m^-^² d^-1^]	6,397.5	2,458.1	6,690.9	5,419.1	2,225.1	1,876.6
total Imports [mg C m^-^² d^-1^]	8,471.0	3,993.1	9,767.4	11,557.7	3,875.7	3,506.7
P_GPP_/B	0.03	0.07	0.09	0.01	0.04	0.04
Total System Throughput [mg C m^-^² d^-1^]	27,228.2	10,576.6	26,205.6	32,139.4	12,303.1	10,722.4
Development Capacity [mg C m^-^² d^-1^ bits]	111,005.2	39,042.7	92,193.7	151,429.6	60,633.2	55,697.3
Ascendency [mg C m^-^² d^-1^ bits]	47,970.3	15,163.1	36,027.3	55,579.7	19,548.0	17,809.1
Overheads [mg C m^-^² d^-1^ bits]	63,034.9	23,879.5	56,166.4	95,850.0	41,085.2	37,888.2
Relative Ascendency [%]	43.2	39.9	39.1	36.7	32.2	32.0
Relative Overheads [%]	56.8	61.1	60.9	63.3	67.8	68.0
Robustness [%]	36.3	36.7	36.7	36.8	36.5	36.5
Flow Diversity [%]	4.1	3.7	3.5	4.7	4.9	5.2
Effective Link-Density	2.2	2.2	2.1	2.8	3.2	3.4
Average Path Length	2.2	1.6	1.7	1.8	2.2	2.1
Finn Cycling Index [%]	2.0	4.0	1.9	1.1	6.4	5.3
Logarithmic Trophic Efficiency [%]	5.7	10.3	5.1	3.8	10.7	6.1
TrophicDepth	3.4	2.7	2.6	3.3	3.0	3.2
Detritivory:Herbivory ratio [D:H]	1: 3.7	1: 1.1	1: 3.2	1: 4.4	1: 1.8	1: 1.9

#### Razor clam field

The razor clam field is a small system with a low amount of available energy shown by the low TST of 10,575.3 mgC m^-2^ d^-1^ (Table 3). The low values for ELD (2.2) and APL (1.7) indicate a simple organization with only few parallel and short pathways. However, the high TE (10.3%) implies that the energy is transferred very efficiently but is only used within few compartments (low APL) which is in accordance with the few functional roles (TD = 2.7). The razor clam field has a high P_GPP_/B value of 0.07 implying an immature state of the system which could be supported by the low values of FD (3.7) and ALP as both values are supposed to increase when the system matures [61].

#### Mud flat

The mud flat is a big and active system with a TST of 26,205.6 mgC m^-2^ d^-1^ (Table 3). It shows a high degree of organization with 39.1%. But, the high P_GPP_/B value indicates an immature state and low levels of TE (5.1%) and ELD (2.1) show that the system is neither efficient nor very robust due to a lack of parallel pathways. Furthermore, the low recycling of 1.9% tends to result in a strong system’s dependency on external imports and the low APL of 1.7 shows that the energy is only used over short pathways. The mud flat therefore appears to be in a stressed and unstable condition [58, 75, 76]. The Lindeman spine shows that only 17.7% of the energy of trophic level I is transported to higher trophic levels (Fig 2). A high amount of energy is therefore stucked at the basis of the food web.

#### Mussel bank

Among the six analyzed habitats the mussel bank provides the highest amount of energy with a total production of 5,371.6mgC m^-2^ d^-1^ (Table 3). It is furthermore the most active system with the highest potential to develop (TST = 32,139.4 mgC m^-2^ d^-1^ and DC = 151,429.6 mgC m^-2^ d^-1^). Furthermore, with a high value of 63.3% OH/DC the system reveals capacities of free energy to cope with perturbations. The mussel bank has the lowest recycling of all six systems with 1.1% resulting in an increased dependency on external imports. The flow structure of the mussel bank is very complex with a high diversity of even flows (FD = 4.7) and several parallel pathways (ELD = 2.8) indicating that the system is resistant in front of perturbations. The mussel bank also has the lowest value for P_GPP_/B with 0.01 indicating a high degree of maturity which could be explained by the stable structure and the long live span mussel banks can reach in the intertidal area [77].

#### Sand flat

The sand flat is a comparatively small system with a TST of 12,302mgC m^-2^ d^-1^ and has a high amount of free energy (OH/DC = 67.8%, Table 3) indicating a high resistance in front of perturbations. This resistance is supported by a complex and diverse flow structure (FD = 4.9) including a lot of parallel pathways with an ELD of 3.2. The system recycles a comparable high amount of energy with an FCI of 6.4% and is therefore largely independent from external energy sources. In accordance with the low P_GPP_/B of 0.04, high values for FD, ELD and FCI could also be an indication for increased system’s maturity [61, 76, 78].

#### Seagrass meadow

Similar to the sand flat, the seagrass meadow has a low TST (10,722.4 mgC m^-2^ d^-1^) with a higher degree of OH/DC than A/DC showing a small system with high capacities of free energy (Table 3). The system reveals the highest FD with 5.2 showing a very complex flow structure with diverse and even flows and a high number of parallel pathways with an ELD of 3.4 indicating an increased resistance in front of perturbations [79]. The high value for FCI (5.3%) shows that the energy is recycled to a large extent implying that the system is independent from external imports. The low P_GPP_/B of 0.04 suggests that the system is in a mature status [61]which is in accordance with its high values for FD, ELD and FCI.

### Comparison between the systems

#### Comparison of the indices

Although the number of compartments differs between the six systems we could not find a linear relationship between the number of compartments and the analyzed system attributes. It is therefore assumed that the systems of this study can be compared with each other as they have the same degree of aggregation. However, the indices DC, A and OH are known to be sensitive to the structure of the model network and therefore not useful on their own for ecological applications [25, 56, 80]. The ratios A/DC and OH/DC are more useful and robust to network construction [7, 29, 56, 80, 81] and therefore more appropriate for comparing the condition and development status of the different systems.

**Fig 3:**
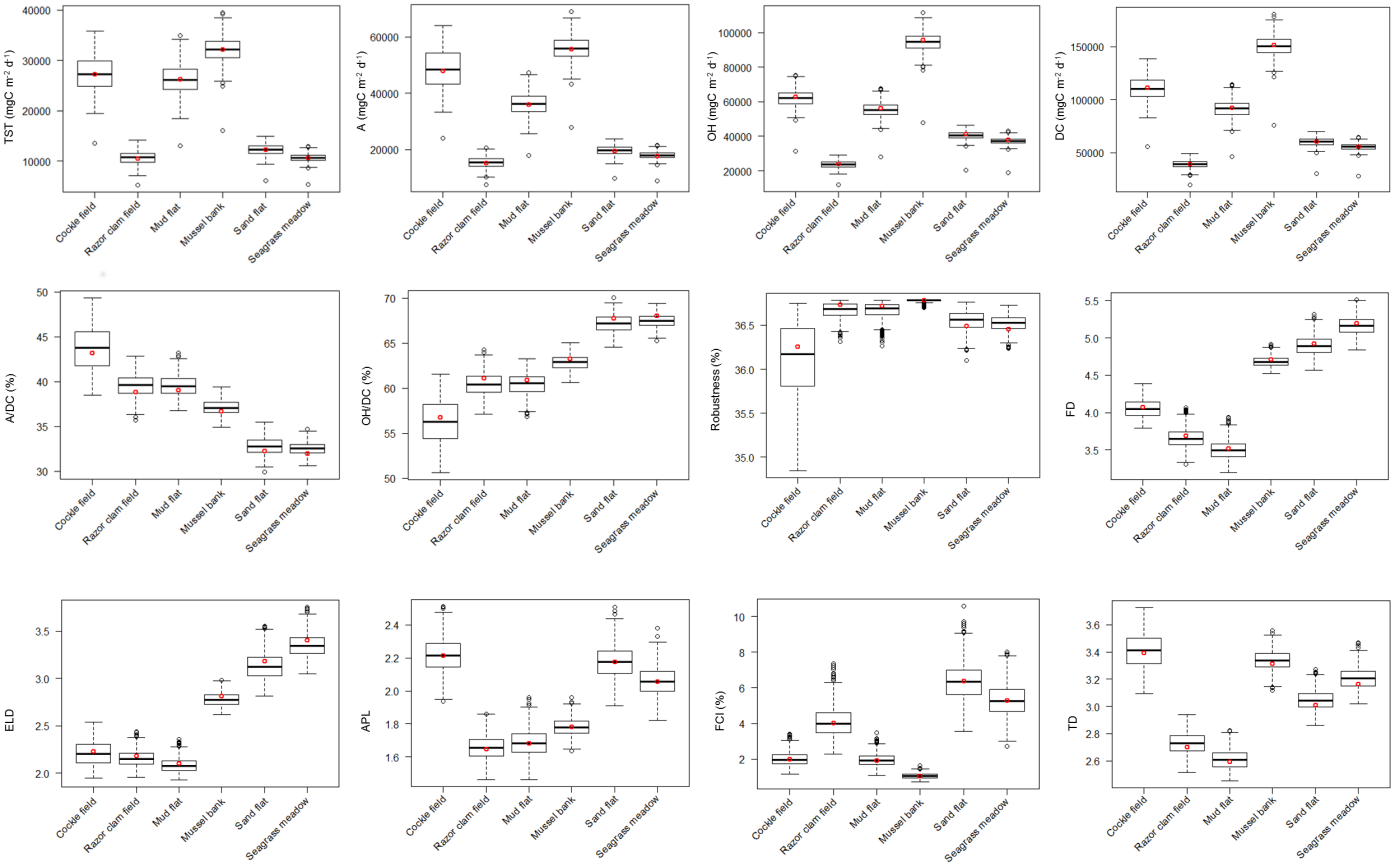
Variation of the indices considered with the uncertainty analysis using 1,000 iterations for each habitat, the initial model is displayed as a red dot.

The uncertainty analysis reveals a wide range of possible solutions for most of the indices in the different habitats (Fig 3). However, there are still sets of indices which differ significantly between some habitats supporting the analyzed results of the initial models. The cockle field, the mud flat and the mussel bank appear to be bigger and more active than the razor clam field, the sand flat and the seagrass meadow indicted by a higher TST. The Ascendency follows the same trend. The cockle field and the mussel bank show furthermore higher values for OH and DC than the razor clam field, the sand flat and the seagrass meadow. The degree of organization A/DC is significantly higher in the cockle field, the razor clam field and the mud flat in comparison to the sand flat and the seagrass meadow which reveal higher values for OH/DC. There are no significant differences in the system attribute robustness. Considering the flow structure, FD and ELD are significantly higher in the mussel bank, the sand flat and the seagrass meadow than in the cockle field, the razor clam field and the mud flat. The APL is higher in the cockle field than in the razor clam field, the mud flat and the mussel bank. The recycling (FCI) is higher in the sand flat and the seagrass meadow than in the cockle field, the mud flat and the mussel bank. The cockle field, the mussel bank and the seagrass meadow also show higher values for TD than the razor clam field and the mud flat.

#### Comparison of system functioning

There are similarities as well as differences between the systems. All six systems reveal a similar value of the robustness index indicating that all six systems are in a good balance between their degree of organization and their amount of free energy. Furthermore, herbivory surpasses detritivory in all six systems. The difference between herbivory and detritivory was the highest in the cockle field and the mussel bank (1: 3.7 and 1: 4.4, respectively) and the lowest in the razor clam field (1: 1.1, Table 3).

Comparing the habitats among each other, there are several similarities between the indices of the cockle field and the mussel bank. Both systems appear to be big and active with a high TST but simultaneously dependent on external imports implied by the low FCI. Also the Lindeman spines of the cockle field and the mussel bank are relatively similar: high external imports support both systems (Fig 2). Trophic efficiencies are comparable in the first four trophic levels but the mussel bank exceeds the trophic efficiency of the cockle field in the upper trophic levels. Additionally, the mussel bank reveals higher values for FD and ELD than the cockle field implying a more complex and redundant flow structure which results in a higher resistance towards disturbances.

The razor clam field is also characterized by a bivalve species but differs in its system structure from the cockle field and the mussel bank. The razor clam field is a small system with a more simple flow structure than the mussel bank (FD and ELD) and less functional roles than the cockle field (TD).

The mud flat is a comparable big system with its high TST but due its simple flow structure with only few parallel pathways and the low recycling the system is probably more sensitive to perturbations than the other intertidal habitats.

The sand flat and the seagrass meadow show similarities as well. Both systems are rather small with a high amount of free energy to cope with perturbations. Furthermore, both systems reveal a high FD implying a complex and even flow structure with several parallel pathways (high ELD) indicating and increased resistance in front of disturbances. Both systems are rather independent from external energy sources due to their high recycling. In terms of the Lindeman spine, exceptional high values are shown in both systems for trophic efficiency on trophic level II with more than 40% (Fig 2).

## Discussion

### Structure and functioning of the systems

#### Similarities between the systems

The six intertidal habitats reveal similarities as well as differences in their food web structure and functioning. Despite the differences in A/DC, the robustness index values of all six systems are very similar indicating that they have a sufficient amount of both, organization and reserves of free energy. Fath [59] hypothesized that an ecological system needs to attain a balance between organization and redundancy to be sustainable. Without this balance a system is predestined to perish [59, 82]. The analyzed six systems appear to be in a good trade-off between their organization and their redundancy implying that they are able to sustain their functions over time [59]. Another similarity between the systems is the high degree of herbivory. Intertidal areas are often characterized by a major role of detritivores in the energy transfer [7, 8, 10] in opposition to herbivory. However, in our six systems, herbivory always exceeds detritivory. The dominance in abundance and biomass of suspension feeders and grazers in the habitats relying on phytoplankton and MPB increase the herbivory strongly resulting in a less important influence of detritivores in all six systems. Furthermore, the high abundance of herbivorous birds feeding on macroalgae and seagrass amplify the difference even more.

#### Cockle field and mussel bank

The cockle field and the mussel bank are both characterized by accumulations of bivalve species which are colonized by various macrobenthic invertebrates and both provide a rich food source for foraging birds. The results of this study show that mussel banks and habitats with similar functioning such as cockle fields are very diverse systems with a high degree in activity and organization but low cycling values and therefore a strong dependency on external imports. This is consistent with the study of Baird, Asmus [10] on mussel banks in the Sylt-Rømø Bight. However, the mussel bank appears to be more resistant than the cockle field. Low values of FD and ELD in the cockle field indicate a low diversity of flows and only few parallel pathways probably caused by a strong reliance of the system on the cockle compartment as a food source for predators. This dependency on a single compartment induces an increased vulnerability to perturbations whereas the higher biodiversity of the mussel bank increased also the system’s resistance. Furthermore, the mussel bank exceeds the efficiency of the cockle field at the higher trophic levels presumably due to the higher abundance of benthic predators such as shore crabs or carnivorous polychaetes which use the lower trophic levels as food sources and being themselves eaten by top predators such as birds. The energy transfer is therefore increased in the food chain.

Although mussel banks as well as cockle fields are rather small-scaled habitats, they both appear to be very important foraging areas for birds. Their high productivity and the rich benthic fauna attract a large variety of bird species. Especially the eider duck (*Somateriamollissima*) is dependent on these habitats as most of its prey consists of mussels and cockles [83]. But also resident bird species (i.e. *Haematopusostralegus*) and migrating waders (i.e. *Limosalapponica*) use these habitats for foraging.

#### Razor clam field

The American razor clam (*Ensisleei*) was introduced to the North Sea in the late 1970s [84, 85] and was discovered as a suitable prey organism by several bird species in recent years [86]. In this study the razor clam field appears to be a small system with indications of perturbation such as the low APL. This is accordance with the location of this habitat in the lower intertidal area which is characterized by harsh abiotic conditions such as intensive current velocities and high sediment mobility. Most of the energy in this habitat is transferred from phytoplankton to razor clams and then in the upper trophic levels to gulls. Perturbations that would affect phytoplankton as the main food source or the razor clam as the dominating organisms could lead to a complete collapse of this system. The latter was already often observed during cold winters or washouts which induced a mass mortality of the razor clam [87]. Natural influences like this make the razor clam system short-lived and could also explain the indications for immaturity.

#### Mud flat

Diverse studies about food web systems of mud flats exist and reveal differences in the functioning of mud flats in combination to their location and environmental circumstances such as fresh water inflow or eutrophication [75]. In the present study, the mud flat was described as a big and active system which might be sensitive to perturbations due to a lack of a complex and redundant flow structure. MPB was one of the major food sources in the mud flat but there was still a high amount of unused MPB production resulting in a decreased trophic efficiency from the first trophic level onwards to the higher trophic levels. Comparable results were observed in the French Brouage mud flat [5]. The system was characterized by a dominant influence of MPB and low values of carbon recycling. High amounts of primary production provide a rich food source for herbivores at lower trophic levels but it was noted that there was the risk of food depletion at higher predator levels. On the contrary, Baird, Asmus [10] described the mud flat of the Sylt-Rømø Bight to be a system characterized by high recycling and great energy reserves to cope with perturbations.

Mud flats are known to be very productive intertidal systems which are of high importance for foraging birds [14]. Waders such as the bar-tailed godwit (*Limosalapponica*) and the Eurasian oystercatcher (*Heamatopusostralegus*) but also the common shellduck (*Tadornatadorna*) and gulls (e.g. *Chroicocephalusridibundus*) were mostly observed feeding on the mud flat. Furthermore, it was the only habitat type where pied avocets (*Recurvirostraavosetta*) were seen. This may be an effect of the feeding modes of this species which are well suited to take up comparatively small prey items in well penetrable sediments. In the Wadden Sea, the population of pied avocets showed an overall decline since 1990 although it was declared to be stable in the federal state of Schleswig-Holstein in the last years [3, 4, 88]. Mud flats appear to be one of the preferred foraging areas of this bird species but the present study shows that the mud flat system might be vulnerable in front of perturbations. These results should be taken into account with respect to protection and management plans concerning bird species such as the pied avocet.

#### Sand flat and seagrass meadow

Sand flats are the most expanded habitat type in the study site. They are often characterized by a high abundance of the lugworm *Arenicola marina*, a preferred food item of several bird species [43]. Seagrass meadows, on the other hand, are shallow water habitats that provide shelter for a diversity of organisms [89]. They are used as a nursery ground for juvenile fish and present a rich food source for herbivorous birds [89, 90]. Although the sand flat and the seagrass meadow are very different in their biological features, we found several similarities in their functioning. They reveal comparable results for their size, their degree of organization, their flow structure (FD and ELD) and in the recycling. Previous work on food webs of sand flats and seagrass meadows of Baird, Asmus [10] already revealed comparable results for both systems in terms of a high FD and a balance between detritivory and herbivory. But the degree of organization was markedly higher in the studies of Baird, Asmus [10]. In case of seagrass beds this might be due to the higher age of the seagrass beds in the Sylt-Rømø Bight compared to the younger and more pioneering type of meadows of the present study site. The TE was markedly higher in both systems of the present study compared to the systems of Baird, Asmus [10] probably caused by a higher bird predation.

Both systems were strongly exploited by a huge number of foraging birds, resulting in an increased trophic efficiency especially on the second and third trophic level. While sand flats are already known to be important feeding grounds for birds, the high abundance of foraging birds on the seagrass meadow is relatively surprising. Former observations indicated that seagrass meadows are of minor importance as a food source for non-herbivorous birds [91] but our results indicate the contrary. Seagrass meadows often occur close to the shore in sheltered areas [92] which can easily be disturbed by human influences such as increased tourism. In this study, the seagrass meadow was isolated and situated further away from the shore and was less influenced by human disturbances. This might explain the high abundance of birds feeding on this habitat in contrast to the formerly observed seagrass meadows in the Sylt-Rømø Bight [91]. But also the long exposure time of the seagrass meadow could play a role. Therefore, it would be interesting to include more seagrass meadows situated in diverse location (i.e. disturbed by human activities or remoted) in further studies to assess their overall importance for foraging shore birds. Furthermore, our results suggest that birds might intensively use seagrass meadows as foraging areas when they are undisturbed environments indicating that conservation measures and management plans should focus on this particular habitat.

### Birds in food web studies

Due to their high mobility birds are very difficult to include in quantitative analyses such as food web studies. Nevertheless, they are very important predators in the intertidal areas and it is strongly recommended to include birds in ecosystem models [43]. Numbers of birds but also their feeding behavior can strongly differ in correlation with the season, water level and time of low tide but also based on the location of the intertidal habitat and its exposure time [93, 94]. It is therefore difficult to draw general conclusions on bird predation from the counts that were done in the present study as the chosen habitats but also the time of counting and the subjective error of the investigator might have biased the results. Bird predation can show high variability from one day to the other and from one sand flat to a neighbored one as birds also react to small-scaled differences [93, 94].

In the uncertainty analysis these circumstances were taken into account by randomly changing flows within the networks. The uncertainty of the system attributes shows different intensities but reveal wide ranges for some indices which could be an indication that the natural variabilities of each compartment could influence the overall functioning of the different systems.

### Comparison with previous studies

Comparisons between different food web studies are usually difficult as the focus of the studies and the aggregation of compartments can differ strongly. This might bias the results of the different network analyses. In the present study we focused on the link between the intertidal benthos communities and birds as top predators. Comparable intertidal models of the Sylt-Rømø Bight [9–13] and the Brouage mud flat [5, 14] are more complete with additional compartments including zooplankton and fish. The model of the Jade Bay [8], does not include higher predator levels such as fish or birds but is very detailed on the macrozoobenthic level with almost each species representing one compartment.

However, there are some noticeable differences between the present models and the earlier analyzed models of the Sylt-Rømø Bight, the Brouage mud flat and the Jade Bay. The first one is the comparatively low recycling in all six habitat types of the present study. This could be either a relic of network construction because unused detritus was assumed to be exported during high tideor a result of the difference in the location of the study area. Keeping excess detritus in the system could result in increased values for FCI [8, 10] as the amount of recycled energy is higher than in systems in which excess detritus is exported. In addition, in the North Sea ENA is often applied in well-studied, enclosed bays and bights with little water exchange. In contrast, the present site was an open system with a direct connection to the open sea that imports regularly a high amount of food for suspension feeders presumably resulting in a less important role of recycling in this area compared to enclosed marine ecosystems.

Comparisons in the food web structure of different habitat types were rarely done before. Baird, Asmus [10] analyzed eight different intertidal systems in the Sylt-Rømø Bight also including mussel banks, seagrass meadows, sand flats and mud flats. Except for the already mentioned differences in cycling and the ratio between detritivory and herbivory, the results of Baird, Asmus [10] for these four habitats matched the ones of the present study.

To increase the comparability of the present study it will be necessary to create a food web model of the whole study site and then analyze the system attributes and their relation to the structure of similar systems. Furthermore, it would be interesting to include compartments such as zooplankton and fish to have a more complete food web which is closer to reality. Such studies could also be used as an important background for management and protection plans in the Wadden Sea. However, habitat diversity appears to be of great importance for the Wadden Sea. Each habitat has its specific characteristics and features and seems to play a different role in the entire Wadden Sea ecosystem.

## Conclusions

In this study we conducted food web analysis for six intertidal habitat types in the Wadden Sea that were known to be important forging areas for coastal bird species. The general structure of the six food webs revealed a good trade-off between the degree of organization and the ability to cope with disturbances in all six systems. However, the systems differ in their detailed features. The cockle field and the mussel bank are big and active systems but with a strong reliance on external phytoplankton input. The razor clam field was shown to be a small system in an immature status. The studied mud flat appeared to be sensitive to perturbations but is still used by a lot of different bird species. The sand flat and the seagrass meadow revealed several similarities in their structure and seem to be in a stable and mature status with a high importance for a large variety of foraging birds.

Our results show that every habitat has its own features and characteristics. Therefore, habitat diversity is an important trait for the function of the Wadden Sea as a whole ecosystem. Every habitat type plays a different role in the heterogeneous mosaic, but it remains unknown to what extend the different habitat types contribute to the whole system. As a next step, it would be necessary to conduct an Ecological Network Analysis of the whole study site to get insight into the complex interactions between the different habitat types and their influence on the whole system structure.

## Supporting information

S1 File SCOR-file of the cockle field. (TXT)

S2 File SCOR-file of the razor clam field. (TXT)

S3 File SCOR-file of the mud flat. (TXT)

S4 File SCOR-file of the mussel bank. (TXT)

S5 File SCOR-file of the sand flat. (TXT)

S6 File SCOR-file of the seagrass meadow. (TXT)
